# In Vivo Cerebral Imaging of Mutant Huntingtin Aggregates Using ^11^C-CHDI-180R PET in a Nonhuman Primate Model of Huntington Disease

**DOI:** 10.2967/jnumed.123.265569

**Published:** 2023-10

**Authors:** Daniele Bertoglio, Alison R. Weiss, William Liguore, Lauren Drew Martin, Theodore Hobbs, John Templon, Sathya Srinivasan, Celia Dominguez, Ignacio Munoz-Sanjuan, Vinod Khetarpal, Jeroen Verhaeghe, Steven Staelens, Jeanne Link, Longbin Liu, Jonathan A. Bard, Jodi L. McBride

**Affiliations:** 1 Bio-Imaging Lab, University of Antwerp, Antwerp, Belgium;; 2 Molecular Imaging Center Antwerp, University of Antwerp, Antwerp, Belgium;; 3 Division of Neuroscience, Oregon National Primate Research Center, Beaverton, Oregon;; 4 Division of Animal Resources and Research Support, Oregon National Primate Research Center, Beaverton, Oregon;; 5 Center for Radiochemistry Research, Oregon Health and Science University, Portland, Oregon;; 6 Integrated Pathology Core, Oregon National Primate Research Center, Beaverton, Oregon;; 7 CHDI Management/CHDI Foundation, Los Angeles, California; and; 8 Department of Behavioral Neuroscience, Oregon Health and Science University, Portland, Oregon

**Keywords:** mHTT, Huntington disease, nonhuman primate, PET, brain

## Abstract

Huntington disease (HD) is a neurodegenerative disorder caused by an expanded polyglutamine (CAG) trinucleotide expansion in the huntingtin (*HTT*) gene that encodes the mutant huntingtin protein (mHTT). Visualization and quantification of cerebral mHTT will provide a proxy for target engagement and a means to evaluate therapeutic interventions aimed at lowering mHTT in the brain. Here, we validated the novel radioligand ^11^C-labeled 6-(5-((5-methoxypyridin-2-yl)methoxy)benzo[d]oxazol-2-yl)-2-methylpyridazin-3(2H)-one (^11^C-CHDI-180R) using PET imaging to quantify cerebral mHTT aggregates in a macaque model of HD. **Methods:** Rhesus macaques received MRI-guided intrastriatal delivery of a mixture of AAV2 and AAV2.retro viral vectors expressing an HTT fragment bearing 85 CAG repeats (85Q, *n* = 5), a control HTT fragment bearing 10 CAG repeats (10Q, *n* = 4), or vector diluent only (phosphate-buffered saline, *n* = 5). Thirty months after surgery, 90-min dynamic PET/CT imaging was used to investigate ^11^C-CHDI-180R brain kinetics, along with serial blood sampling to measure input function and stability of the radioligand. The total volume of distribution was calculated using a 2-tissue-compartment model as well as Logan graphical analysis for regional quantification. Immunostaining for mHTT was performed to corroborate the in vivo findings. **Results:** ^11^C-CHDI-180R displayed good metabolic stability (51.4% ± 4.0% parent in plasma at 60 min after injection). Regional time–activity curves displayed rapid uptake and reversible binding, which were described by a 2-tissue-compartment model. Logan graphical analysis was associated with the 2-tissue-compartment model (*r*^2^ = 0.96, *P* < 0.0001) and used to generate parametric volume of distribution maps. Compared with controls, animals administered the 85Q fragment exhibited significantly increased ^11^C-CHDI-180R binding in several cortical and subcortical brain regions (group effect, *P* < 0.0001). No difference in ^11^C-CHDI-180R binding was observed between buffer and 10Q animals. The presence of mHTT aggregates in the 85Q animals was confirmed histologically. **Conclusion:** We validated ^11^C-CHDI-180R as a radioligand to visualize and quantify mHTT aggregated species in a HD macaque model. These findings corroborate our previous work in rodent HD models and show that ^11^C-CHDI-180R is a promising tool to assess the mHTT aggregate load and the efficacy of therapeutic strategies.

## Visual Abstract

**Figure:**
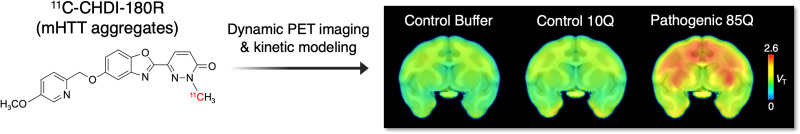

Huntington disease (HD) is an autosomal dominant neurodegenerative disorder caused by an expanded polyglutamine (CAG) repeat in exon 1 of the huntingtin (*HTT*) gene (1,2). This mutated gene encodes the mutant huntingtin protein (mHTT), which is cleaved into N-terminal fragments, accumulates into intracellular inclusion bodies, and plays a pathophysiologic role in neurodegeneration (3–6). These neuropathologic features are known to severely impact the caudate and putamen (collectively, the striatum) but are also evident in several other cortical and subcortical brain regions (3,7,8). Several promising therapeutic candidates aimed at lowering mHTT in the brain have been developed and are undergoing clinical evaluation (9–11). In this context, quantifying the brainwide spatial distribution of mHTT protein with region-level resolution offers a proxy for target engagement and evaluation of the regional pharmacologic effects of such therapeutic interventions (9). Toward this goal, we evaluated ^11^C-labeled 6-(5-((5-methoxypyridin-2-yl)methoxy)benzo[d]oxazol-2-yl)-2-methylpyridazin-3(2H)-one (^11^C-CHDI-180R) as a ligand specific for mHTT aggregates that is cell- and brain-permeable and has high affinity (1–3 nM) and selectivity (12–14). Recently, we reported that ^11^C-CHDI-180R PET imaging can noninvasively quantify mHTT brain aggregates in HD mouse models and offers insight into the time-, dose-, and region-specific pharmacodynamic activity in distinct mHTT-lowering interventional paradigms (15).

Application of ^11^C-CHDI-180R in larger animal models of HD would be beneficial in monitoring future HTT-lowering efficacy studies. We recently created an adeno-associated virus (AAV)–mediated rhesus macaque model of HD wherein mHTT is expressed throughout the caudate, putamen, and several cortical and subcortical brain regions, in a pattern similar to that observed in people with HD (16). Immunohistochemical studies using monoclonal antibodies for (m)HTT in this model have verified the formation of EM48- and 2B4-positive mHTT aggregates in these same brain regions (16,17). Furthermore, this macaque model shows working memory impairment and motor dysfunction (chorea, dystonia, tremor, incoordination) and develops structural and functional corticostriatal changes including mild atrophy, increased white matter diffusivity, reduced cerebral glucose metabolism, and altered striatal D_2/3_ receptor density (17,18).

Here, we investigated the novel radioligand ^11^C-CHDI-180R using PET imaging at 30 mo after surgery in the HD macaque model and controls. Specifically, we assessed the plasma profile of ^11^C-CHDI-180R, examined kinetic models for the volume of distribution (*V*_T_) estimation, and explored its capability for quantification of mHTT aggregates and correspondence with behavioral phenotypes.

## MATERIALS AND METHODS

### Animals

Fourteen rhesus macaques (aged 7–14 y; weight, 5.5–13.6 kg; 10 female and 4 male) were used in this study. All animals received MRI-guided stereotactic injections of a 1:1 mixture of AAV2.retro and AAV2 at a titer of 1e12 vg/mL (2e12 vg/mL combined) expressing a fragment of mHTT with 85 CAG repeats (85Q), a control fragment of HTT with 10 CAG repeats (10Q), or a buffered saline injection w/F-Pluronic (BASF Corp.) (5 with AAV2:2retro-HTT85Q, 4 with AAV2:2retro-HTT10Q, and 5 with phosphate-buffered saline). Viral vectors were infused into the caudate and putamen (2 injections per region per hemisphere; total of 8 injections per animal), for a total volume of 330 μL per hemisphere, as reported in detail previously (17).

The guidelines specified in the National Institutes of Health Guide for the Care and Use of Laboratory Animals were strictly followed. All experimental procedures were approved by the Institutional Animal Care and Use Committee and the Institutional Biosafety Committee at the Oregon National Primate Research Center and Oregon Health and Science University.

### Tracer Radiosynthesis

^11^C-CHDI-180R was synthesized using an automated module (TRACERlab FXC; GE Healthcare) by adapting a method we previously described (15). The CHDI-180R precursor was radiolabeled by mixing the precursor (0.5–1.2 mg) with dimethylsulfoxide (100 ± 50 μL) and 4–10 mg of cesium carbonate (Cs_2_CO_3_). Then, ^11^C-CH_3_I was synthesized using a TRACERlab FX2-MEI box and bubbled into the vented reaction vial. The reaction was heated at 60°C ± 5°C for 1 min, after which 0.9 mL of the preparative mobile phase (0.10 M ammonium formate:acetonitrile; 60:40 v/v) was added to the reaction and the reaction mixture was injected onto a semipreparative high-performance liquid chromatography (HPLC) column (BetaBasic C18 7.6-mm outer diameter × 250-mm length; Thermo Scientific) at a flow rate of 1–1.5 mL/min. The radioactive product was passed through a sterilizing 0.2-μm filter in a volume of 1–1.5 mL to an empty sterile vial. The product radioactivity was assayed and diluted with sterile, preservative-free 0.9% saline. The mass of ^11^C-CHDI-180R in the product was analyzed using HPLC mass spectrometry with ultraviolet and radiation detection to determine product radiochemical purity, chemical purity, injected mass, and identity. ^11^C-CHDI-180R was synthesized with a radiochemical purity of more than 99% and a molar activity of 1,412 ± 556 GBq/μmol (mean ± SEM) at the end of synthesis.

### Dynamic PET/CT Acquisition

Dynamic 90-min PET/CT imaging was performed using a Discovery MI 710 PET/CT imaging system (GE Healthcare). The animals were anesthetized with ketamine HCl (10–15 mg/kg intramuscularly), intubated, and maintained on 1%–2% isoflurane in oxygen. A saphenous intravenous catheter was placed for ligand administration, and a saphenous artery catheter, for blood collection. Before each PET scan, an 8-s CT scan was acquired using 100 kV and 50 mA for coregistration, attenuation, and scatter correction. A bolus of radioligand (131.3 ± 46.9 MBq) was injected manually over a 30-s interval immediately after the start of the 90-min dynamic PET scan. The resulting total injected mass was 0.055 ± 0.03 μg/kg. Detailed information on the animal and dosing parameters is provided in Supplemental Table 1 (supplemental materials are available at http://jnm.snmjournals.org). No significant difference in any of the dosing parameters was observed among the experimental groups. Dynamic PET data were acquired in list-mode format and were subsequently reconstructed into 28 frames of increasing length (4 × 15 s, 4 × 30 s, 4 × 60 s, 4 × 120 s, 9 × 300 s, and 3 × 600 s) using the GE Healthcare software (Q.clear technology). Normalization, scatter, dead time, and CT-based attenuation corrections were applied. PET image frames were reconstructed on a 120 × 100 × 52 grid with 1.823 × 1.823 × 2.780 mm voxels.

### Input Function and Radiometabolite Analysis

In parallel to the PET acquisition, serial arterial blood samples were obtained to calculate arterial input functions and correct for the presence of plasma radiometabolites (15). Sixteen blood samples (1 mL each) at 0.25, 0.5, 0.75, 1, 1.5, 2, 2.5, 3, 5, 8, 15, 30, 45, 60, 75, and 90 min after injection were collected in 3-mL heparinized syringes. At each time point, radioactivity was measured in 300 μL of whole blood and 300 μL of plasma in a cross-calibrated γ-counter (Wizard2; PerkinElmer) and used to calculate the plasma–to–whole-blood ratio. An additional 1.0 mL of arterial blood was collected at 5, 15, 30, 45, 60, 75, and 90 min after injection to measure the parent fraction during the scan and correct the plasma input function for radiometabolites. After separation via a centrifuge (×3,000 relative centrifugal force for 5 min), 100 μL of deproteinated plasma supernatant were loaded onto a preconditioned reverse-phase HPLC column (Phenomenex Luna C18 (2) 5-μm HPLC column [250 × 4.6 mm] plus Phenomenex security guard precolumn) and eluted with sodium acetate buffer (0.05 M, pH 5.5) and acetonitrile (55:45 v/v) for 12 min at a flow rate of 1 mL/min. After elution, 2-min HPLC fractions were collected and measured in the γ-counter for quantification of the radiometabolite and parent fractions. The radioactivity associated with each peak was expressed as a percentage of the total area of the peaks based on the radiochromatograms to allow determination of the percentage contribution of the parent ligand to the total radioactivity signal at each sampling time.

With PMOD software (version 4.2; PMOD Technologies), individual metabolite-corrected plasma arterial input function for kinetic modeling of the PET data were obtained by correcting individual input functions by parent fraction values fitted with a sigmoid curve, as well as correcting for the plasma-to-whole blood ratios. The plasma free fraction was assessed (Supplemental Fig. 1) but not considered for quantification given the limited accuracy when measured through ultrafiltration.

### Image Processing and Analysis

PET data were analyzed and processed using PMOD software. Spatial normalization of PET images to ONPRC18 MRI template space (19) was performed using individual T2-weighted MR images collected as part of an ongoing longitudinal study with the same animals (17). Once the dynamic PET images were normalized to the template space, the volumes of interest defined by the template were used to extract regional time–activity curves for cerebral gray and white matter regions. This animal model displays only a mild atrophy (∼5% volume change over time) in the striatum and cortex; therefore, partial volume correction was not needed (17).

In line with our previous observation in mice (15), ^11^C-CHDI-180R kinetics were described by a 2-tissue-compartment model or Logan plot method (20). Thus, quantification of the total *V*_T_ was achieved using a 2-tissue-compartment model with blood volume fraction (*V*_B_) fixed at 4%, after testing 3.5% and 4.5%, based on fitting of the model. The linear phase for the Logan plot was determined from the curve fitting based on 10% maximal error. For 1 animal (ID12), blood data were not available for the first 2.5 min; therefore, 2-tissue-compartment model data were not available for this animal.

To evaluate the time stability of the *V*_T_ estimates, PET data were reanalyzed by excluding the last 10 min of the PET acquisition from 90 to 40 min. The *V*_T_ estimates obtained using the 90-min PET acquisition were considered the reference outcome with which values from shorter acquisitions were compared. *V*_T_ estimates were considered acceptable if the mean percentage difference compared with the 90-min PET acquisition was below 10%, with an interindividual SD lower than 5%.

Parametric images were generated with PMOD software using the pixelwise modeling tool (PXMOD) through voxel-based graphical analysis (Logan plot). Parametric maps are not smoothed and are represented as group averages and overlaid onto the study-specific MRI brain template for anatomic reference. Voxelwise statistical analysis of the parametric *V*_T_ maps was performed using statistical parametric mapping (SPM, version 12; Wellcome Department of Imaging Neuroscience). Statistical T-maps were calculated for a peak voxel threshold of *P* = 0.01 (uncorrected) and a cluster of at least 10 voxels (k > 10). First, we confirmed the lack of significantly increased or decreased voxels between control groups (buffer and 10Q); next, we combined the control groups and compared them with the 85Q. The 85Q group did not display any reduced voxels; therefore, only clusters of increased binding are reported.

### Behavioral Measures

As part of a previous longitudinal experiment, all 14 animals involved in this study completed a behavioral assessment of motor and cognitive function a few weeks before ^11^C-CHDI-180R scanning (17). Briefly, motor phenotypes were assessed with a nonhuman-primate (NHP)–specific rating scale modified from the Unified Huntington’s Disease Rating Scale, and cognitive capacities were measured using the 3-Choice Spatial Delayed Response task. Full methodologic details on these tasks are included in a previous work (17).

### Immunostaining

Brain sections were immunohistochemically stained as previously described (16). Briefly, 40-μm-thick sections were incubated with the 2B4 antibody against (m)HTT (1-82aa, MAB5492, 1:1,000; Millipore) and a goat antimouse secondary antibody (BA-9200, 1:500; Vector Laboratories). The signal was developed using a standard Vectastain ABC kit (PK6100; Vector Laboratories) with subsequent incubation in 3,3′-diaminobenzidine (112080050; Sigma) and nickel (II) sulfate hexahydrate (N4882; Sigma) intensification. Images magnified to ×4 and ×20 were captured on an Olympus BX51 microscope with an Olympus DP72 camera controlled by the Olympus cellSens program from representative cases.

### Statistical Analysis

A 2-way ANOVA with Holm–Šidák multiple comparison testing was applied to compare ^11^C-CHDI-180R scan parameters and *V*_T_ among experimental groups in the different brain structures. Pearson correlation tests were used to compute all correlations. Statistical analyses were performed using Prism (version 9; GraphPad) and SPSS (version 28.0; IBM). Data are represented as mean ± SD. All tests were 2-tailed, and significance was set at a *P* value of less than 0.05.

## RESULTS

### Blood Analysis

**FIGURE 1.**
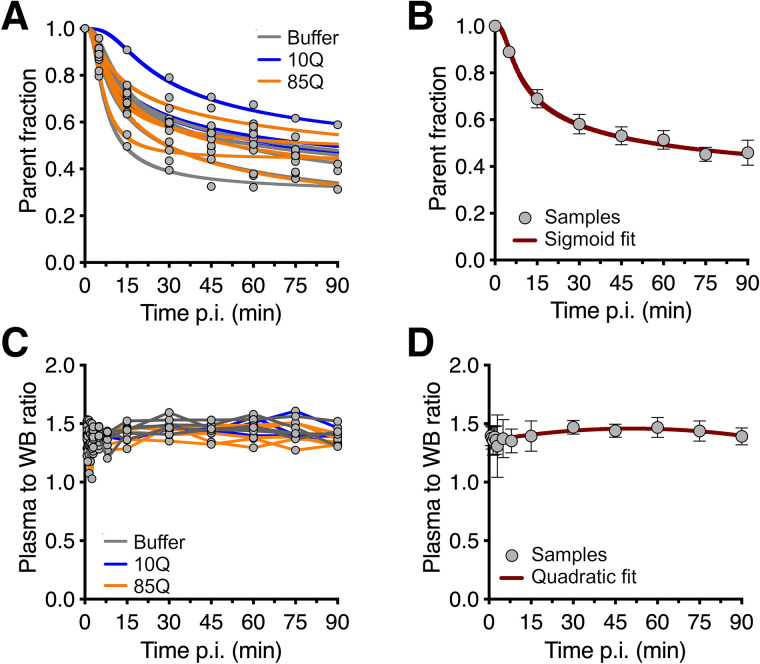
Blood analysis of ^11^C-CHDI-180R in NHPs. Parent fraction profile in plasma for individual subjects (A) as well as group profile (B). Plasma–to–whole-blood ratio for individual subjects (C) as well as group profile (D) over time after intravenous injection of ^11^C-CHDI-180R. Not all samples were available at 90 min because of radioactive decay. Data in B and D are mean ± SD (*n* = 14). p.i. = after injection; WB = whole blood.

Serial blood samples were collected from animals in all groups during each scan to derive the individual metabolite-corrected plasma arterial input function for kinetic modeling of the PET data. No apparent difference in radiometabolite profile of the radioligand was observed among the 3 experimental groups. After intravenous injection, the overall parent fraction (^11^C-CHDI-180R) appeared to decrease slowly with time (Fig. 1A), accounting for 51.4% ± 4.0% of the total plasma radioactivity at 60 min after injection (Fig. 1B); the decline profile was described by a sigmoid fit. With radio-HPLC, only polar radiometabolites could be identified, suggesting low potential for brain-penetrant species. Finally, the plasma–to–whole-blood ratio did not show any apparent change over time (Fig. 1C), although, at the group level, it was best described by a quadratic fit (Fig. 1D). The plasma free fraction was 44.2% ± 9.3% and did not differ among the 3 experimental groups (Supplemental Fig. 1).

### Description of ^11^C-CHDI-180R by a 2-Tissue-Compartment Model

**FIGURE 2.**
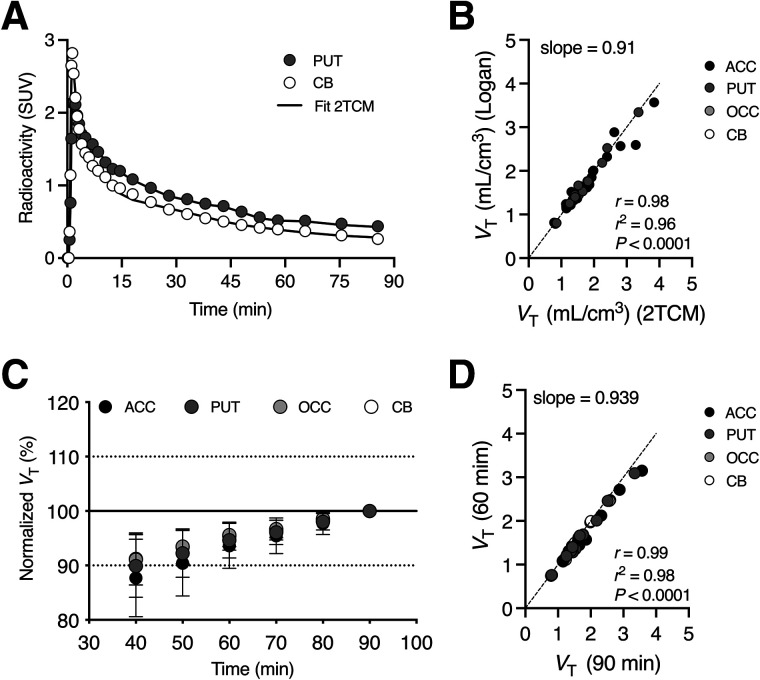
Kinetic modeling of ^11^C-CHDI-180R in NHPs (*n* = 13). (A) Two-tissue-compartment model describes SUV time–activity curves of ^11^C-CHDI-180R in regions with both high (putamen) and low (cerebellum) cerebral uptake in 85Q animal. (B) Comparison of ^11^C-CHDI-180R *V*_T_ estimates using 2-tissue-compartment model and Logan plot for ^11^C-CHDI-180R quantification in 4 different brain regions (anterior cingulate cortex and occipital cortex). (C) *V*_T_ estimates using Logan plot with different scan durations normalized to values obtained with 90-min acquisition. (D) Comparison of ^11^C-CHDI-180R *V*_T_ estimates using Logan plot based on 90- or 60-min scan acquisition. 2TCM = 2-tissue-compartment model; ACC = anterior cingulate cortex; CB = cerebellum; OCC = occipital cortex; PUT = putamen.

We evaluated ^11^C-CHDI-180R kinetics in animals from all groups by performing 90-min dynamic PET acquisitions after intravenous injection. Representative brain regions with high (putamen) and low (cerebellum) ^11^C-CHDI-180R uptake during the 90-min acquisition are reported in Figure 2A. ^11^C-CHDI-180R displayed rapid cerebral uptake, peaking within 3 min after injection, with fast washout. Reversible kinetics were described by a 2-tissue-compartment model (Fig. 2A). A description of microparameters and goodness of fit is available in Supplemental Table 2. In line with the rodent findings (15), the Logan graphical analysis was a valid alternative to obtain *V*_T_ estimates based on 4 different brain regions (*r*^2^ = 0.96, *P* < 0.0001) (Fig. 2B).

In a second exploratory analysis, data from the 90-min scans were reanalyzed, assessing scanning intervals ranging from 90 to 40 min. The time stability of *V*_T_ estimates based on Logan graphical analysis indicated an underestimation of the outcome parameter with shortening of the scan acquisition (Fig. 2C). Nonetheless, *V*_T_ estimates based on a 60-min acquisition were comparable to the values obtained with the 90-min acquisition with a deviation of −5.3% ± 3.2%. This was also confirmed by the strong correlation in *V*_T_ measures obtained with the different scan durations (slope = 0.939, *r*^2^ = 0.98, *P* < 0.0001) (Fig. 2D), indicating that a scan acquisition of a minimum of 60 min is reliable for estimation of ^11^C-CHDI-180R *V*_T_.

### Detection of mHTT in an NHP Model of HD by ^11^C-CHDI-180R PET Imaging

**FIGURE 3.**
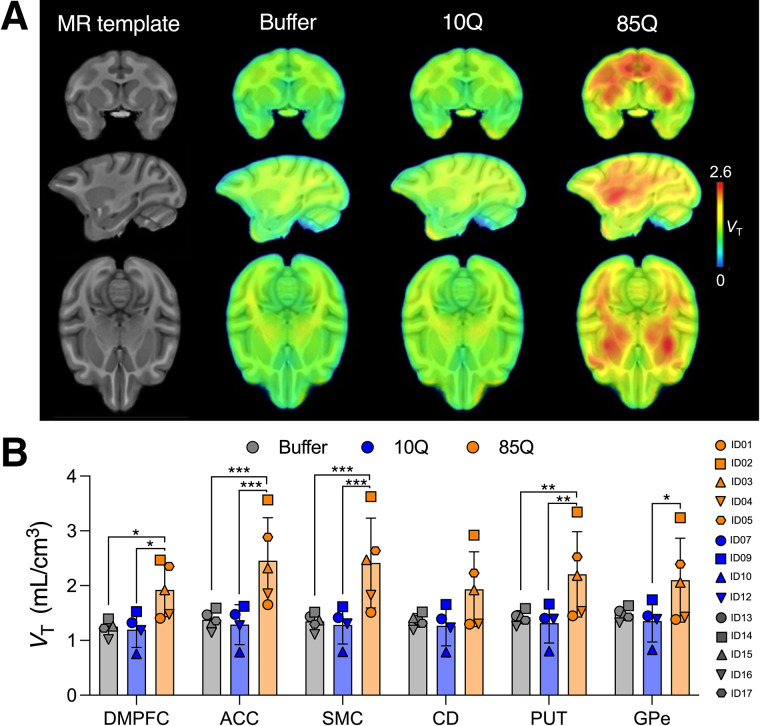
PET imaging using ^11^C-CHDI-180R in NHPs. (A) Averaged parametric *V*_T_ maps in coronal (top), sagittal (middle), and axial (bottom) planes are overlaid onto ONPRC18 MRI T1-weighted rhesus macaque brain template. Animals in 85Q group show higher ^11^C-CHDI-180R *V*_T_ than animals in control groups (buffer or 10Q). (B) Quantification of ^11^C-CHDI-180R *V*_T_ in relevant brain structures. 85Q group displayed significantly higher ^11^C-CHDI-180R *V*_T_ than control groups (buffer or 10Q). No statistical difference between buffer and 10Q was observed. (Buffer, *n* = 5; 10Q, *n* = 4; 85Q, *n* = 5.) ACC = anterior cingulate cortex; CD = caudate; DMPFC = dorsomedial prefrontal cortex; GPe = external segment of globus pallidus; PUT = putamen; SMC = supplemental motor cortex. *P < 0.05. **P < 0.01. ***P < 0.001.

We previously demonstrated that intrastriatal delivery of AAV2 or AAV2.retro expressing 85Q leads to transduction of the caudate and putamen and of several other brain structures, resulting in the expression of mHTT and the formation of 2B4- and EM48-postitive mHTT aggregates in these brain regions (16,17). Accordingly, evident binding in the parametric ^11^C-CHDI-180R *V*_T_ map for the 85Q group was visible in several cerebral structures compared with the buffer or 10Q-injected control groups (Fig. 3A). Regional analysis of ^11^C-CHDI-180R *V*_T_ revealed a statistically significant main effect of group (*F*_(2,748)_ = 165.1, *P* < 0.0001), with no effect of brain region (*F*_(67,748)_ = 1.226, *P* = 0.1127) or interaction (group × brain region) (*F*_(134,748)_ = 0.205, *P* > 0.99). Post hoc group analysis indicated a statistically significant difference between 85Q and control groups (buffer or 10Q) in several brain regions (Fig. 3B), whereas no differences were observed between buffer and 10Q in any volumes of interest, as expected given the lack of the target in the control groups. Group *V*_T_ values and post hoc statistical comparisons for all gray matter structures are shown in Supplemental Figure 2 and reported in Supplemental Table 3, respectively.

**FIGURE 4.**
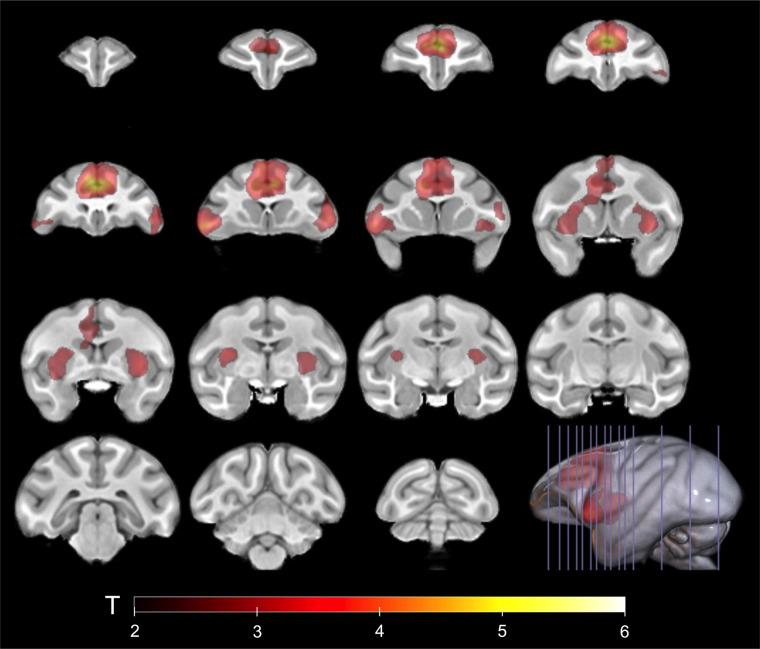
Increased voxelwise ^11^C-CHDI-180R binding in 85Q NHPs compared with control groups combined. Clusters of significantly increased ^11^C-CHDI-180R *V*_T_ in 85Q animals are overlaid onto ONPRC18 MRI template (threshold of *P* < 0.01, cluster size > 10). Scale bar represents T scores. (Buffer, *n* = 5; 10Q, *n* = 4; 85Q, *n* = 5.)

Next, we used a voxelwise analysis to investigate the subregional binding of ^11^C-CHDI-180R. The voxelwise analysis detected significantly increased clusters of ^11^C-CHDI-180R in 85Q animals compared with controls (Fig. 4) in the brain regions reported in the volume-of-interest–based analysis. Specifically, the voxel-based approach also identified subregional areas of increased ^11^C-CHDI-180R binding, including the head of the caudate, the dorsolateral and ventrolateral prefrontal cortices, the dorsal premotor cortex, and the insular cortex (Fig. 4).

**FIGURE 5.**
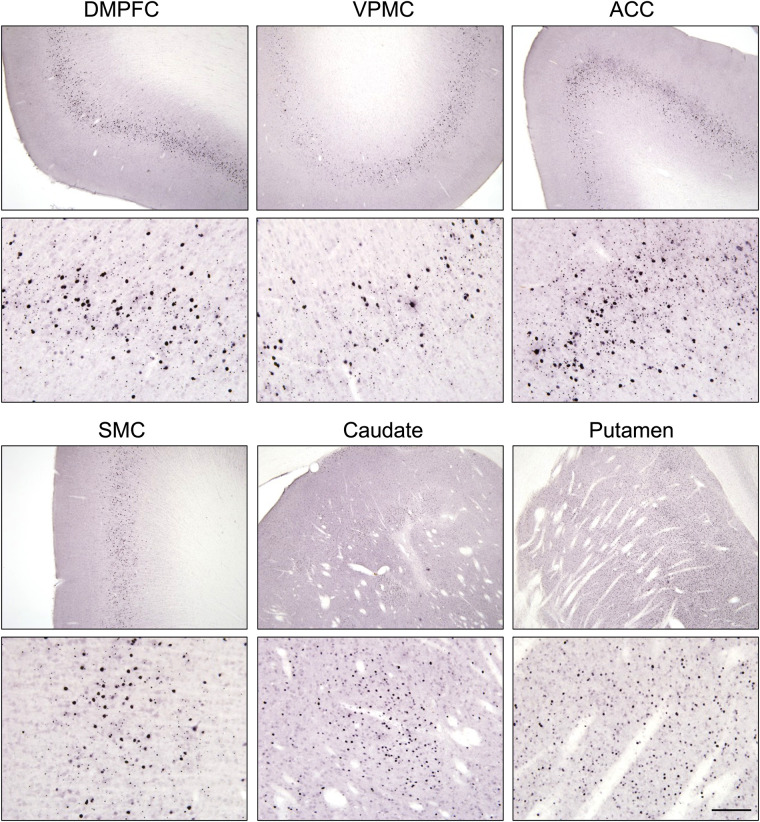
2B4 immunostaining in 85Q NHPs showed agreement with areas of increased ^11^C-CHDI-180R *V*_T_. Images at ×4 (top) and ×20 (bottom) of mHTT aggregates detected by 2B4 staining in same brain regions show increased ^11^C-CHDI-180R *V*_T_ in 85Q primates, including regions distal from injection sites (caudate and putamen) such as dorsal medial prefrontal cortex, ventral premotor cortex, anterior cingulate cortex, and supplemental motor cortex. Scale bar = 100 μm. ACC = anterior cingulate cortex; DMPFC = dorsal medial prefrontal cortex; SMC = supplemental motor cortex; VPMC = ventral premotor cortex.

To confirm the expression of (m)HTT and formation of mHTT aggregates in transduced brain regions, 2B4 immunohistochemistry was performed on tissue collected at necropsy shortly after the conclusion of these PET studies (∼31 mo after surgery). Figure 5 shows the presence of soluble and aggregated mHTT in representative cases from 85Q animals in regions of significantly increased ^11^C-CHDI-180R *V*_T_ (Supplemental Fig. 3 shows individual cases and quantification). No (m)HTT aggregates were observed in the control groups (buffer and 10Q). Increased ^11^C-CHDI-180R *V*_T_ in white matter structures was also detected both at the regional level (Supplemental Fig. 4; Supplemental Table 4) and at the voxel level (Fig. 5). Histologic investigation of white matter confirmed the presence of 2B4-positive (m)HTT aggregates in these brain structures (Supplemental Fig. 5), supporting the specificity of ^11^C-CHDI-180R binding. Although mHTT accumulation in white matter was not anticipated, the formation of mHTT aggregates appears to be limited to the regions in and adjacent to the needle tracts created during AAV-vector delivery.

Finally, as part of a previous longitudinal study, the same animals completed behavioral assessments of motor and cognitive function a few weeks before ^11^C-CHDI-180R scanning (17). We inquired whether behavioral deficits were associated with ^11^C-CHDI-180R binding. Analyses revealed that there was a significant correlation between behavioral scores and *V*_T_ values in several cortical and subcortical regions, such that animals with greater behavioral deficits (higher neurologic rating scores and lower working memory scores) had higher levels of ^11^C-CHDI-180R binding (Table 1).

**TABLE 1.** Correlations Between Behavioral Scores and ^11^C-CHDI-180R *V*_T_ Values in Gray Matter Structures

Gray matter structure	Neurologic rating		Working memory	
	Correlation	*P*	Correlation	*P*
Dorsolateral prefrontal cortex	0.522	0.056	−0.6120	0.034*
Ventrolateral prefrontal cortex	0.533	0.050*	−0.6220	0.031*
Orbitofrontal cortex	0.406	0.150	−0.5770	0.050*
Ventromedial prefrontal cortex	0.443	0.112	−0.5550	0.0610
Dorsomedial prefrontal cortex	0.672	0.008^†^	−0.6630	0.019*
Anterior cingulate cortex	0.703	0.005^†^	−0.6370	0.026*
Dorsal premotor cortex	0.504	0.066	−0.5870	0.045*
Ventral premotor cortex	0.594	0.025*	−0.5980	0.040*
Supplemental motor cortex	0.667	0.009^†^	−0.6130	0.034*
Primary motor cortex	0.451	0.106	−0.5550	0.061
Superior temporal cortex	0.419	0.136	−0.5450	0.067
Inferior temporal cortex	0.406	0.150	−0.5360	0.072
Rhinal cortex	0.374	0.187	−0.5270	0.078
Insular cortex	0.566	0.035*	−0.5680	0.054
Somatosensory cortex	0.432	0.123	−0.5540	0.062
Parietal cortex	0.388	0.170	−0.5350	0.073
Posterior cingulate cortex	0.46	0.098	−0.5650	0.056
Occipital cortex	0.375	0.187	−0.5160	0.086
Caudate	0.51	0.063	−0.5720	0.052
Putamen	0.589	0.027*	−0.5930	0.042*
Internal globus pallidus	0.458	0.100	−0.5310	0.076
External globus pallidus	0.511	0.062	−0.5570	0.060
Lateral thalamus	0.402	0.155	−0.5210	0.082
Medial thalamus	0.387	0.172	−0.5060	0.093
Hippocampus	0.434	0.121	−0.5390	0.071
Amygdala	0.473	0.088	−0.5520	0.063
Substantia nigra	0.43	0.125	−0.5740	0.051
Cerebellum	0.423	0.132	−0.5370	0.072

* *P* < 0.05.

† *P* < 0.01.

Higher neurologic rating scores, and lower working memory scores, are associated with greater impairment. Correlations are 2-tailed Pearson. (Buffer, *n* = 5; 10Q, *n* = 4; 85Q, *n* = 5.)

## DISCUSSION

Various new therapeutic approaches for HD focus on the modulation of mHTT levels (9,11,21). To determine whether these approaches are achieving target engagement in the relevant brain structures, and whether they are leading to lowering mHTT levels in vivo, the development of noninvasive quantification of mHTT is needed (12–14,22,23). We recently demonstrated that ^11^C-CHDI-180R PET imaging is able to quantify mHTT aggregates in HD mouse models (15). Here, we offer evidence that ^11^C-CHDI-180R is applicable in a large NHP model of HD bearing mHTT aggregates and behavioral phenotypes (16,17).

In contrast to previous preclinical mouse studies (15), analysis of the plasma profile of NHPs revealed the formation of at least one radiometabolite more polar than ^11^C-CHDI-180R. This radiometabolite polarity, together with the measured brain time–activity curves, suggested no evidence of brain-penetrant radiometabolite species. This is important validation for the use of this ligand in humans and lays the foundation for future studies to use this NHP model to test the efficacy of therapeutic interventions designed to lower mHTT in the brain.

The results of both regional and voxelwise quantification of ^11^C-CHDI-180R revealed significantly increased binding in several brain regions in the 85Q group compared with the buffer and 10Q control groups, suggesting specific binding to mHTT aggregates. It was previously demonstrated that the intrastriatal delivery of a mixture of AAV2 and AAV2.retro leads to transduction in the caudate and putamen as well as several cortical structures (as a result of the retrograde transport capability of AAV2.retro), forming detectable mHTT aggregates in all of these regions (16,17). This distribution profile is closely recapitulated by the binding pattern of ^11^C-CHDI-180R PET imaging, with the putamen, anterior cingulate cortex, and supplemental motor cortex representing the most affected gray matter structures in this model (16,17). Importantly, we found an association between the increased binding pattern of ^11^C-CHDI-180R PET imaging and the behavioral assessments of motor and cognitive function in the 85Q group.

Compared with genetically engineered animal models of HD, in which mHTT expression tends to be uniform throughout the brain, viral vector-based models often result in mHTT expression in specific targeted brain regions or subregions. Accordingly, voxel-based analyses in the AAV2:AAV2retro-based macaque HD model identified significant ^11^C-CHDI-180R binding in subregions of several brain structures, some of which were not identified using the region-of-interest-based approach. This finding suggests that voxel-based analyses may also be useful in human HD studies, in which mHTT aggregates are more concentrated in subregions of certain structures, such as the lateral putamen and in deeper layers of the cerebral cortex (5,24). Additionally, should PET imaging be used in future studies to demonstrate target engagement of HTT-lowering therapeutics, voxel-based analysis may be able to discern HTT changes when therapeutic constructs reach only subregions of brain structures after delivery.

We performed a postmortem histologic investigation to confirm the formation of 2B4-positive mHTT aggregates in brain regions that showed significantly increased ^11^C-CHDI-180R *V*_T_. However, an important limitation of such a comparison is that this radioligand does not bind to large mHTT species, as detected by the common antibodies used for histologic assessment, such as EM48 and 2B4. We have previously demonstrated binding to mHTT-derived fibrils and to mHTT aggregates expressed in mouse HD models and human HD samples, whose precise state has yet to be defined (12,13,15). Given that radioligand and antibodies do not recognize the same mHTT species, a direct association between readouts is not possible. Therefore, histologic analysis was performed to confirm the presence of mHTT aggregates in relevant brain regions.

Accordingly, in 85Q animals, 2B4-positive aggregates were detected in the regions of injection (caudate and putamen) and in several cortical gray matter structures that send afferent projections to the striatum. The anterior-to-posterior cortical gradient observed on immunostaining aligns with a similar distribution observed with PET imaging and recapitulates the previously described distribution of the AAV2:AAV2.retro viral vector mixture (16). We also detected increased ^11^C-CHDI-180R binding in the white matter of 85Q-treated animals, a finding that was not anticipated given the lack of white matter binding observed during in vitro ^3^H-CHDI-180 autoradiography studies in mouse models and human postmortem tissue (12,13,15). Histologic evaluation shows 2B4-positive mHTT aggregates in white matter tracts in, and near, visible needle tracts. This finding is not surprising, given that the infusion pump was run while the needle was lowered into the caudate and putamen to maintain positive pressure, prevent infusate backflow, and prevent tissue damage.

Autoradiography has been used in genetic mouse models and human HD brain tissue samples, in combination with immunohistochemistry, to confirm regional and subregional binding of CHDI-180R. Although tissue was not available in the current study to perform autoradiography, pilot studies on 4 adult macaques injected with either AAV2retro-85Q (left hemisphere) and diluent (right hemisphere) or AAV2retro-10Q (left hemisphere) and diluent (right hemisphere) used autoradiography to demonstrate specific binding in the putamen, globus pallidus, and cortex of 85Q-injected animals but not in 10Q- or diluent-injected controls.

## CONCLUSION

Our study demonstrated the validity of ^11^C-CHDI-180R as a reliable radioligand to visualize and quantify the region-specific distribution of mHTT aggregated species in the HD macaque model with high spatial resolution. These findings corroborate our previous work in rodent HD models and further support ongoing investigation of ^11^C-CHDI-180R as a pharmacodynamic biomarker in HD.

## DISCLOSURE

Daniele Bertoglio was supported by the Research Foundation Flanders (FWO) (1229721N and K201222N) and the University of Antwerp (FFB210050). Daniele Bertoglio, Jeroen Verhaeghe, and Steven Staelens are members of the µNeuro Research Centre of Excellence at the University of Antwerp. Alison Weiss was supported by a postdoctoral fellowship (NIH/NIA T32AG055378). Jodi McBride is supported by NIH grant P51OD011092, and the creation of the NHP model of HD used in this project was supported by NIH grant NS099136. Microscopy was supported in part by the Imaging and Morphology Core at the Oregon National Primate Research Center (S10OD025002-01). Radiolabeling was done using the Oregon Health and Science University Center for Radiochemistry Research Core Facility. This work was funded by the CHDI Foundation, Inc. (https://chdifoundation.org/). Vinod Khetarpal, Longbin Liu, Celia Dominguez, Ignacio Munoz-Sanjuan, and Jonathan Bard are employed by CHDI Management, Inc., as advisors to CHDI Foundation, Inc., a privately funded nonprofit biomedical research organization dedicated exclusively to collaboratively developing therapeutics that improve the lives of those affected by HD. No other potential conflict of interest relevant to this article was reported.

**KEY POINTS**

**QUESTION:** Can we quantify mHTT aggregates in an NHP model of HD in vivo?

**PERTINENT FINDINGS:** In this study, we demonstrated that the regional accumulation of mHTT aggregates in the brain can be quantified using PET imaging with ^11^C-CHDI-180R in a macaque model of HD.

**IMPLICATIONS FOR PATIENT CARE:** ^11^C-CHDI-180R PET imaging quantifies the regional accumulation of mHTT aggregates and offers a promising new avenue to examine the efficacy of mHTT-lowering therapeutic strategies.
